# Transcatheter arterial embolization for gastrointestinal bleeding: Clinical outcomes and prognostic factors predicting mortality

**DOI:** 10.1097/MD.0000000000029342

**Published:** 2022-08-05

**Authors:** Shinhaeng Lee, Taehwan Kim, Seung Chul Han, Haeyong Pak, Han Ho Jeon

**Affiliations:** a Department of Internal Medicine, National Health Insurance Service Ilsan Hospital, Goyang, Korea; b Department of Radiology, National Health Insurance Service Ilsan Hospital, Goyang, Korea; c Institute of Health Insurance & Clinical Research, National Health Insurance Service Ilsan Hospital, Goyang, Korea.

**Keywords:** embolization, gastrointestinal hemorrhage, prognosis, therapeutic

## Abstract

We evaluated clinical outcome and prognostic factors predicting mortality of transcatheter arterial embolization (TAE) for acute gastrointestinal (GI) bleeding. Fifty-nine patients (42 men, 17 women; mean age 66.1 ± 17.0) who underwent 59 TAE procedures for GI bleeding during 2013–2018 were retrospectively evaluated. Clinical outcomes included technical success, adverse events, and rebleeding and mortality rate within 30 days. The technical success rate was 100%. Angiography showed contrast extravasation in 41 (69.5%) patients and indirect signs of bleeding in 16 (27.1%) patients. Two (3.4%) patients underwent prophylactic embolization. TAE-related adverse events occurred in 7 (11.9%) patients; adverse events were more common for mid GI or lower GI bleeding than for upper GI bleeding (22.6% vs 0%, *P* = 0.007). Rebleeding within 30 days was observed in 22 (37.3%) patients after TAE. Coagulopathy was a prognostic factor for rebleeding (odds ratio [OR] = 3.53, 95% confidence interval 1.07–11.67, *P* = .038). Mortality within 30 days occurred in 11 (18.6%) patients. Coagulopathy (OR = 24, 95% confidence interval 2.56–225.32, *P* = .005) was an independent prognostic factor for mortality within 30 days. TAE is an effective, safe, and potentially lifesaving procedure for GI bleeding. If possible, coagulopathy should be corrected before TAE as it may reduce rebleeding and mortality.

## 1. Introduction

Acute gastrointestinal (GI) bleeding, which can occur because of various causes, such as GI ulcers, diverticulosis, malignancy, angiodysplasia, and trauma, may have various clinical courses and symptoms depending on the site and cause of bleeding. Endoscopic intervention is the primary diagnostic and treatment method for GI bleeding. However, it is difficult to accurately diagnose or treat GI bleeding if there is food, feces, or a blood clot in the GI tract in an emergency. In addition, evaluation of the small intestine is generally limited. Patients with GI bleeding who are unresponsive to pharmacologic therapy, in whom endoscopic intervention has failed, or who are unable to undergo endoscopy, angiographic intervention, or surgical intervention require measures to control of bleeding.^[1,2]^ In the case of upper GI bleeding, if bleeding occurs again after successful endoscopic hemostasis, it is recommended to try endoscopic treatment again rather than performing surgery or transcatheter arterial embolization (TAE). However, if endoscopic treatment fails, TAE is a reasonable therapeutic choice in management. Because TAE shows significant reductions in complications and hospital stay with no difference in mortality compared with surgery.^[3]^ In the case of lower GI bleeding, TAE should be considered in patients who do not respond adequately to hemodynamic resuscitation treatment and cannot perform bowel preparation and urgent colonoscopy. Computed tomography angiography should be considered when diagnostic tests are required to identify bleeding sites before TAE. In general, surgery for acute lower GI bleeding should be considered only after other treatment options have failed.^[4]^ However, for a large number of patients with acute GI bleeding, surgical intervention may also be limited due to various comorbidities; the majority are referred for angiography intervention. TAE has been performed since first reported by Rocsh and Dotter in 1972. With the development of embolization technology and embolization materials, TAE has been effective at controlling GI bleeding and decreasing mortality.^[5–7]^ The efficacy and safety of TAE have been reviewed in several studies of upper GI bleeding, but the results did not show sufficient control of lower GI bleeding.^[8,9]^ In addition, only a few studies have reported on the clinical outcomes of TAE and prognostic factors predicting mortality in upper and lower GI bleeding.

This study aimed to evaluate clinical outcomes, including technical success, adverse events, and rebleeding and mortality rate within 30 days of TAE, of patients with acute GI bleeding when endoscopic intervention had failed or was not feasible. We present the following article in accordance with the STROBE reporting checklist.

## 2. Patients and Methods

### 2.1. Patients

This was a retrospective study performed at National Health Insurance Ilsan hospital. We reviewed the medical records of patients with acute GI bleeding who underwent TAE from January 2013 to December 2018. Contrast-enhanced abdominal pelvic computed tomography (CT) was performed in most patients to identify bleeding site and cause and perform anatomical analysis. Bleeding site was identified either by endoscopy or CT finding before TAE. GI bleeding was classified into 3 subtypes. Bleeding above the ampulla of Vater was defined as upper GI bleeding, bleeding in the small intestine anywhere from the ampulla of Vater to the terminal ileum was defined as mid-GI bleeding, and colonic bleeding was defined as lower GI bleeding.^[10]^ Inclusion criteria were

1. older than 18 years of age and
2. acute non-variceal GI bleeding either refractory to pharmacologic or endoscopic intervention or perceived to be insufficiently controlled by pharmacologic or endoscopic intervention.

Patients with variceal bleeding and bleeding into the abdominal peritoneal or retroperitoneal space were excluded. This retrospective study was approved by the institutional review board at our institution, and informed consent was waived.

### 2.2. TAE procedure

TAE procedures were performed in the interventional radiology suite. All patients underwent TAE via a puncture to the right or left common femoral artery followed by an angiogram. For patients with upper GI bleeding, the celiac artery and the superior mesenteric artery were selected using a 5 Fr angiographic catheter (COOK, Bloomington, Ind.); then, we confirmed the overall anatomy of the blood vessel and the presence of bleeding. We then coaxially inserted a 2–2.4 Fr microcatheter and performed super-selective angiography on the bleeding site. We assessed the degree of bleeding and corresponding vessels and surrounding anatomical structures through super-selective angiography, which was used to select suitable embolic materials. The superior mesenteric artery and inferior mesenteric artery were selected for patients with lower GI bleeding. The internal iliac artery was additionally examined in patients with rectal and sigmoid colon hemorrhage. Angiography was considered positive when it showed either a direct angiographic sign of active GI bleeding (contrast extravasation) or indirect signs of bleeding.^[9,11]^ Embolization was performed as selectively as possible. If angiography was negative for active bleeding, prophylactic or empiric embolization was performed based on a referral from a physician. The embolic agent was selected according to the anatomical condition of the bleeding vessel, the degree of bleeding, access and selection of the micro-angiographic conduit, and the preference of the radiologist. Post-embolization angiography was also performed.

### 2.3. Measured outcomes and definition

Follow-up data were available for all patients. Data on events were collected by review of the electronic medical records of patients. The primary outcomes of our study were procedure technical success and rebleeding rates within the 30 day from the date of TAE. The secondary outcomes were TAE-related adverse events and survival rates. Technical success was defined as an immediate complete angiographic occlusion of all target vessels contributing to the area of hemorrhage.^[5]^ Coagulopathy was defined as international normalized ratio (INR) greater than 1.5, partial thromboplastin time (PTT) longer than 45 second, or a platelet count less than 80,000/mL.^[12,13]^ Clinical failure of TAE was defined as rebleeding within the 30-day follow-up period. Rebleeding was defined as a subsequent bleeding event with clinical bleeding symptoms such as hematemesis, melena, hematochezia, or a decrease in the hemoglobin level of > 2 g/dL. Procedure-related adverse events were classified according to the Society of Interventional Radiology Standards of Practice Committee classification of complication.^[14]^ Patient survival was analyzed until 30 days from the date of TAE and the mortality cause was categorized as hemorrhage-specific and others, which were not directly attributable to GI bleeding but to other potential causes, such as respiratory failure, acute myocardial infraction, or acute stroke or underlying diseases.

### 2.4. Statistical analysis

A sample size of 40 achieves 75% powder to detect a difference between the group proportions of 0.4000 using the two-sided T-test. The significance level of the test was targeted at 0.0500. The significance level actually achieved by this design is 0.0502.

Descriptive statistics were used to characterize the demographics of the study populations. Continuous variables were analyzed using *t*-test. Categorical variables were analyzed using Chi-Squared or Fisher exact test. To identify clinical factors related to rebleeding and mortality, logistic regression analysis was performed. Variables with *P* value <.05 in univariate analysis were included in the multivariate logistic analysis (stepwise regression). A *P* value of <.05 was considered statistically significant. All statistical analyses were performed using SPSS software version 23 (IBM Corp., Armonk, New York).

## 3. Result

### 3.1. Patient characteristics

Fifty-nine patients underwent 59 angiography and embolization procedures. The mean age of patients was 66.1 ± 17.0 years and 17 (28.8%) were women. A total of 41 patients underwent contrast-enhanced abdominal pelvic CT or CT angiography. The median period between CT and TAE was 7 hours (range, 1–91 hours). Twenty-eight of the 59 (47.5%) patients had upper GI bleeding, while the remaining 31 (52.5%) experienced mid GI or lower GI bleeding (Table 1). More than half of patients had a high surgical risk associated with aging and comorbidities; 38/59 (64.4%) patients were older than 65 years, and 34/59 (57.6%) patients had at least 2 comorbidities. Additionally, 13/59 (22%) patients were taking antiplatelet agents or anticoagulants and 22/59 (37.3%) patients had coagulopathy status before TAE. Patients showed various clinical symptoms related to acute GI bleeding, and hematochezia (52.5%) was the most prevalent symptom. Furthermore, 45/59 (76.3%) patients had shock status before TAE. The mean number of packed red blood cells transfused before embolization was 6.0 ± 4.7.

**Table 1:** Patient demographics (n = 59).

Variable	
Age (years)	66.10 ± 0 17.0
Sex (male: female)	42 (71.2)/ 17 (28.8)
In-patient/ ER patient	23 (39)/ 36 (61)
Comorbidities	
Diabetes mellitus	15 (25.4)
Hypertension	35 (59.3)
Heart failure	10 (16.9)
Ischemic heart disease	12 (20.3)
Cerebrovascular disease	7 (11.9)
Malignancy	13 (22)
Peptic ulcer disease	17 (28.8)
Coagulopathy*	22 (37.3)
Medication†	13 (22)
Liver cirrhosis	7 (11.9)
Renal failure	13 (22)
Initial presentation	
Melena	11 (18.6)
Hematemesis	4 (6.7)
Melena + hematemesis	3 (5.2)
Hematochezia	31 (52.5)
Hematochezia + hematemesis	2 (3.4)
Others	8 (13.6)
Shock	45 (76.3)
Hemoglobin0 <0 9.0	7 (11.9)
Bleeding site	
UGI	28 (47.5)
Duodenal ulcer	9 (15.3)
Gastric ulcer	7 (11.9)
Dieulafoy lesion	1 (1.7)
Malignancy	4 (6.7)
Diverticular disease	1 (1.7)
Esophageal tear	1 (1.7)
Unknown cause	5 (8.5)
MGI	21 (35.6)
Pseudoaneurysm	5 (8.5)
Dieulafoy lesion	1 (1.7)
Malignancy	2 (3.4)
HSP	1 (1.7)
Jejunal ulcer	1 (1.7)
Aneurysm	1 (1.7)
Unknown cause	10 (16.9)
LGI	10 (16.9)
Angiodysplasia	1 (1.7)
Malignancy	1 (1.7)
Diverticular disease	2 (3.4)
Colorectal ulcer	2 (3.4)
Unknown cause	4 (6.7)
PRBC unit total transfusion before embolization	6.00 ± 0 4.7

### 3.2. TAE procedure outcomes

Details of the TAE procedures are summarized in Table 2. Thirty-nine of 59 (66.1%) patients underwent emergent TAE (within ≤24 hours after acute bleeding onset). Embolization was performed in 57 (96.6%) patients based on the angiography results, which were direct contrast extravasation (41/59, 69.5%) or indirect signs of bleeding (16/59, 27.1%). Two (3.4%) patients had no definite evidence of bleeding on angiography, but the artery supplying the suspected bleeding site was embolized prophylactically. Single-artery embolization was performed in 53 (89.8%) patients, and more 2 arteries were embolized in 6 (10.2%) patients. Multiple embolic agents (15/59, 25.4%) were used. N-butyl cyanoacrylate (NBCA) glue (44/59, 74.6%) was the most frequently used embolic agent, either alone (30/59, 50.9%) or in combination with other embolic agents (14/59, 23.7%).

**Table 2:** Summary of embolization performed on patients with gastrointestinal bleeding.

Variables	
Urgency of embolization	
Emergent (≤24 h)	39 (66.1)
Urgent (>24 h, ≤7 d)	20 (33.9)
Rationale for embolization	
Contrast extravasation	41 (69.5)
Indirect signs of bleeding	16 (27.1)
Prophylactic or empiric	2 (3.4)
Arteries embolized ≥2 territories embolization	6 (10.2)
Embolic agents	
Coil	10 (16.9)
Gelfoam	4 (6.8)
NBCA glue	30 (50.9)
NBCA glue + coil	10 (16.9)
NBCA glue + gelfoam	2 (3.4)
Coil + PVA	1 (1.7)
NBCA glue + PVA	1 (1.7)
NBCA glue + Coil + PVA	1 (1.7)

The patients undergoing TAE procedures were divided into 2 groups, according to the site of bleeding: upper and mid GI or lower GI bleeding groups. Embolization was technically successful in all patients, having a technical success rate of 100% (Table 3). Adverse events occurred in 7 (11.9%) patients, and all these adverse events occurred in patients with mid GI or lower GI bleeding. Two of these patients underwent artery dissection during the procedure. Five patients were confirmed to have developed post-TAE ischemia. Three of these 5 patients developed bowel infarction; the other 2 patients did not develop any further ischemic complications. Outcomes after TAE were compared between the 2 groups. Although technical success, rebleeding rate, and mortality rate were not significantly different between the 2 groups, the proportion of adverse events was significantly higher in the mid GI or lower GI bleeding group (*P* = .007). Rebleeding occurred in 22 patients (37.3%) after TAE. Patients with recurrent bleeding were treated as follows: 8 patients underwent surgery, and 1 patient underwent endoscopic intervention. Thirteen of these patients received only conservative treatment for bleeding. More active treatment was not desired by these patients or family members. Mortality occurred in 11 patients (18.6%): 6 (54.5%) had a hemorrhage-specific death, and the other deaths (45.5%) were not directly attributable to GI bleeding but other potential causes or underlying diseases.

**Table 3:** Post-embolization result.

Variable	All (59)	UGI (28)	MGI or LGI (31)	*P* value
Technical success	59 (100)	28 (100)	31 (100)	
Adverse events	7 (11.9)	0 (0)	7 (22.6)	.007
Rebleeding (≤30 d)	22 (37.3)	10 (35.7)	12 (38.7)	.812
Mortality rate (≤30 d)	11 (18.6)	6 (21.4)	5 (16.1)	.602
Hemorrhage-specific	6 (54.5)	3 (50.0)	3 (60)	1.0

### 3.3. Clinical factors of rebleeding and mortality after TAE

The results of univariate analysis of the clinical factors for rebleeding and mortality after TAE are presented in Tables 4 and 5, respectively. In univariate analysis, rebleeding was associated with renal failure (*P* = .047), coagulopathy (*P* = .009), and massive transfusion (*P* = .047). Overall, coagulopathy was more likely to occur with recurrent bleeding (odds ratio [OR] = 3.53; 95% confidence interval: 10.7–11.67; *P* = .038). However, malignant bleeding, embolization emergency, bleeding site, embolization territories, and multiple embolic agents showed no effect on rebleeding outcome. In univariate analysis, mortality within 30 days was associated with liver cirrhosis (*P* = .013) and coagulopathy (*P* = .002). Coagulopathy (OR = 24; 95% confidence interval: 2.56–225.32; *P* = .005) was the only factor that was associated independently with mortality in the multivariate analysis. Rebleeding did not affect the mortality within 30 days. Similarly, malignant bleeding, embolization emergency, bleeding site, embolization territories, and multiple embolic agents showed no effect on mortality outcome.

**Table 4:** Clinical factors of rebleeding within 30 days.

	Univariate analysis			Multivariate analysis		
Variable	Clinical success (n0 =0 37)	Rebleeding (n0 =0 22)	*P* value	Odds ratio	95% CI	*P* value
Age (years)	64.50 ± 0 18.0	68.60 ± 0 15.2	.371			
Male	26 (70.3)	16 (72.7)	.840			
Liver cirrhosis	2 (5.4)	5 (22.7)	.065			
Renal failure (CRF or ESRD)	5 (13.5)	8 (36.4)	.047	2.79	0.69–11.21	.149
Malignant bleeding	5 (13.5)	2 (9.1)	.614			
Coagulopathy*	9 (24.3)	13 (59.1)	.009	3.53	1.07–11.67	.038
Medication†	8 (21.6)	5 (22.7)	.921			
Shock	25 (67.6)	20 (90.9)	.056			
Embolization time (>24 h)	12 (32.4)	8 (36.4)	.758			
Bleeding site (MGI or LGI vs UGI)	19 (51.4)	12 (54.5)	.812			
≥2 territories embolization	2 (5.4)	4 (18.2)	.137			
Multiple embolic agents	12 (32.4)	3 (13.6)	.119			
Massive transfusion‡	5 (13.5)	8 (36.4)	.047	2.36	0.58–9.64	.223

**Table 5:** Clinical factors of mortality within 30 days.

	Univariate analysis			Multivariate analysis		
Variable	Survival (n0 =0 48)	Mortality (n0 =0 11)	*P*-value	Odds ratio	95% CI	*P*-value
Age (years)	66.60 ± 0 17.2	63.80 ± 0 17.0	.991			
Male	35 (72.9)	7 (63.6)	.542			
Liver cirrhosis	3 (6.3)	4 (36.4)	.013	2.00	0.32–12.33	.455
Renal failure (CRF and ESRD)	11 (22.9)	2 (18.2)	.747			
Malignant bleeding	5 (10.4)	2 (18.2)	.478			
Coagulopathy*	12 (25)	10 (90.9)	.002	24.00	2.56–225.32	.005
Medication†	12 (25)	1 (9.1)	.274			
Shock	35 (72.9)	10 (90.9)	.232			
Embolization time (>24 h)	16 (33.3)	4 (36.4)	.848			
Bleeding site (MGI or LGI vs UGI)	26 (54.2)	5 (45.5)	.603			
≥ 2 territories embolization	3 (6.3)	3 (27.3)	.056			
Multiple embolic agents	12 (25)	3 (27.3)	.876			
Rebleeding (≤30 days)	15 (31.3)	7 (63.6)	.054			
Massive transfusion‡	9 (18.8)	4 (36.4)	.213			

## 4. Discussion

In this retrospective analysis of 59 patients, despite most patients having high surgical risk associated with aging and comorbidities, TAE effectively controlled both upper and mid GI or lower GI bleeding with high technical success (100%) rates and clinical success (62.7%) rates. Many previous studies have reported technical success rates as high as 90% to 100%.^[11,15–17]^ The results of this study are comparable to those of other series and recommended success thresholds. The causes of the technical failure have been reported as vascular complex anatomy, vascular spasm, and stenosis of the bleeding artery. However, the overall success rate of TAE is very high and TAE has become an excellent approach to control various types of GI bleeding. The most important limitation of TAE is the occurrence of rebleeding. The clinical success rate (no rebleeding within 30 days) in this study was 62.7% (64.3% for upper GI bleeding and 61.3% for mid GI or lower GI bleeding). The rebleeding (37.3%) rate in the present study is comparable to the reported recurrent bleeding rate in other studies.^[9,11,15–17]^ The low clinical success rate compared to the technical success rate is thought to have been caused by various factors, such as rebleeding caused by incomplete embolization, rebleeding of other lesions in the surrounding vessels other than the embolized vessel, and rebleeding due to ischemia after embolization or coagulopathy, even after immediate treatment of GI bleeding after TAE. Therefore, careful follow-up is necessary even after the technical success of embolization. In addition, we identified that coagulopathy was a significant contributor to an increased risk of rebleeding, consistent with previous other studies.^[11,12,15,18]^ This result highlights the need for detailed correction of coagulopathy in patients with GI bleeding.

Adverse events such as injection site hematoma, arterial dissection, and contrast-related complications can occur in 0% to 10% of TAE procedures. Severe adverse events are known to occur in less than about 2% of patients.^[19]^ In this study, there were 2 artery dissections during super selection of the bleeding site. The incidence of post-TAE ischemia or infarction, which is a typical adverse event, is reported to be 0% to 25%, and GI perforation occurred in1.8%.^[6,11,12,15,17]^ The incidence of adverse events of post-TAE in this study was 11.9%, similar to previous studies. All adverse events such as post-TAE ischemia or infraction occurred only in the lower GI bleeding group. There was no post-TAE ischemia in the upper GI tract with sufficient collateral blood supply, but post-TAE ischemia occurred in the lower GI tract with insufficient collateral blood supply, showing a similar result as a previous study.^[20]^ Due to this lack of collateral blood supply, it is thought that all 3 cases of infarction after TAE confirmed in this study are likely to be linked to the lower GI tract. All three infarctions were embolized with NBCA glue. These infarctions were the result of approximately 5 to 6 NBCA glue castings, which showed a clinical progression of about 2 or 3 days after embolism. Additional surgical treatment was required in all patients, and stable clinical progress was observed in all patients after surgery. However, selection of the best embolic agent is still controversial. A variety of embolic materials can be used successfully by experienced radiologists. Recently, good results were achieved with NBCA glue, indicating that it is a safe and effective embolization material for GI bleedings.^[17,21]^ This material is especially useful against massive bleeding that requires emergent hemostasis. However, using NBCA glue requires training and considerable experience, due to the risk of post-TAE infarction and glue reflux to other surrounding vessels. Previous studies have reported that more than 3 embolizations of the vasa recta increase the risk of adverse events such as infraction.^[22–24]^ This observation was confirmed in 3 patients with infraction in this study. Therefore, in patients with GI bleeding, especially lower GI bleeding, further research is needed to prevent post-TAE ischemia or infarction by performing super selection of the bleeding vessel and minimizing strait vessel embolization during embolization using NBCA glue.

The overall morality within 30 days in this study was 18.6% among all patients. The mortality rate in our study cohort is consistent with that reported in prior studies.^[11,17,25,26]^ Many factors have been reported to contribute to post-TAE mortality, including older age, sepsis, recent major surgery, multiple comorbidities, malignancy, massive blood transfusions, coagulopathy, and rebleeding. Our study showed that only coagulopathy was associated with a 24-time increased odds of 30-day mortality. Previous studies have also showed a strong correlation between coagulopathy and mortality after TAE.^[11,18,25]^

The coagulopathy was a significant risk factor for rebleeding and mortality in this study. In patients with GI bleeding, if hemostasis is achieved after endoscopic treatment, we try to maintain a hemoglobin concentration >7.0 g/dL, platelet count >50,000/mL, and prothrombin time (PT) or PTT < 1.5 times normal. However, if bleeding persists after endoscopic treatment or TAE, we target a hemoglobin concentration >8.0 g/dL for transfusion. If more than 6 units of packed RBCs were transfused, balanced transfusion (a 1:1:1 ratio of fresh frozen plasma, platelet concentrate, and red blood cells) was performed. The optimal target INR for endoscopic treatment to effective and safe has yet determined. In the previous study, endoscopic treatment was reported to be as effective in patients who taking warfarin (after correction INR level of 1.5–2.5) as in control.^[27]^ Active treatment like transfusion generally also recommend when bleeding is accompanied by PT or PTT >1.5 times normal and thrombocytopenia with platelet count <50,000–100,000/mL in perioperative bleeding patients.^[28]^ Conversely, there is no data exist on the safety and efficacy of TAE in GI bleeding patients without previous correction of coagulopathy. In the above-mentioned studies,^[11,12,15,18,25]^ coagulopathy was reported to be a poor clinical outcomes, but in this study, about 40% of patients had coagulopathy status before TAE. Therefore, it is considered that strict coagulopathy correction is needed for patients receiving TAE with acute GI bleeding.

There are several limitations in our study. First, this was a retrospective study over 6 years, which resulted in a lack of consistent strategies for the diagnosis and treatment for patients with acute GI bleeding. Second, we could not compare the efficacy of the different embolic agents because of the relatively small number of patients. Third, since this was a single-center study, further research is needed to generalize the results of this study.

In conclusion, this study reveals that TAE is an effective and potentially life-saving method to manage non-variceal GI bleeding when endoscopic hemostasis therapy is unsuccessful or not feasible. Every effort should be made to correct coagulopathy before TAE because coagulopathy is a significant risk factor for both rebleeding and mortality.

## Author contributions

Formal analysis: Haeyong Pak.

Supervisions: Taehwan Kim, Seung Chul Han.

Writing – original draft: Shinhaeng Lee.

Writing – review & editing: Han Ho Jeon.

## Footnotes

* Defined as international normalized ratio (INR) greater than 1.5, partial thromboplastin time (PTT) longer than 45 second, or platelet count less than 80,000/ml.

† Antiplatelet agents or anticoagulants.

* Defined as international normalized ratio (INR) greater than 1.5, partial thromboplastin time (PTT) longer than 45 second, or platelet count less than 80,000/mL.

† Antiplatelet agents or anticoagulants.

‡≥ 10 packed red blood cell units transfusion before embolization.

* Defined as international normalized ratio (INR) greater than 1.5, partial thromboplastin time (PTT) longer than 45 s, or platelet count less than 80,000/mL.

† Antiplatelet agents or anticoagulants.

‡≥ 10 packed red blood cell units transfusion before embolization.
